# Examining the Effects of Temperature on Recombination in Wheat

**DOI:** 10.3389/fpls.2020.00230

**Published:** 2020-03-11

**Authors:** Alexander Coulton, Amanda J. Burridge, Keith J. Edwards

**Affiliations:** School of Biological Sciences, University of Bristol, Bristol, United Kingdom

**Keywords:** recombination, temperature, meiosis, wheat, genotyping

## Abstract

Meiotic recombination plays a crucial role in the generation of new varieties. The effectiveness of recombination is limited by the distribution of crossover events, which in wheat and many other crops is skewed toward the distal regions of the chromosomes. Whole-genome sequencing of wheat has revealed that there are numerous important genes in the pericentromeric regions, which are inaccessible to manipulation due to the lack of crossover events. Studies in barley have shown that the distribution of recombination events can be shifted toward the centromeres by increasing temperature during meiosis. Here we present an analysis of the effects of temperature on the distribution and frequency of recombination events in wheat. Our data show that although increased temperature during meiosis does cause an inward shift in recombination distribution for some chromosomes, its overall utility is limited, with many genes remaining highly linked.

## Introduction

Meiosis is a specialized type of cell division that leads to the production of haploid gametes. A key feature of meiosis is the process of recombination, where parental genetic material is shuffled together to create chimeric chromosomes, somewhat akin to shuffling a pack of playing cards. Recombination is crucial to the evolution of species, facilitating the spread of beneficial combinations of alleles whilst allowing unfavorable ones to be reduced in the population (Wilkins and Holliday, 2009). In addition to its role in the formation of natural populations, meiosis is also exploited in agriculture, where breeders cross different varieties of crops or animals together to produce offspring with a mixture of both parental phenotypic traits (Acquaah, 2007). This principle has previously been exploited to produce dramatic yield increases in staple food crops, such as during the green revolution, in which short-stemmed Japanese wheat varieties were crossed with high-yielding American varieties (Russell, 1985). This resulted in plants that were less susceptible to lodging (Rajikumara, 2008).

Whilst recombination has previously been harnessed to great effect, its utility is limited in important staple food crops such as wheat (Saintenac et al., 2009; Choulet et al., 2014), maize (He et al., 2017), and barley (Higgins et al., 2014). The distribution of recombination events in these crops is strongly skewed toward the distal ends of the chromosomes, with little to no crossovers (COs) occurring in the region surrounding the centromere, known as the pericentromeric region (Zelkowski et al., 2019). This contrasts with *Arabidopsis*, in which the distribution of COs is much more uniform, with only the centromeric region showing highly reduced numbers of COs (Giraut et al., 2011; Choi et al., 2013). With the advent of a chromosome-level genome assembly for wheat (IWGSC et al., 2018), it is now known that genes are distributed somewhat evenly along the chromosomes, with many potentially important genes being found in the pericentromeric region. The limitation in recombination distribution therefore creates a problem for breeders, as it is not possible to break up large central linkage blocks. A central area of current research in crops is to examine the cause of this skewed recombination distribution and, following this, to try and implement measures to induce recombination in the pericentromeric region (Higgins et al., 2012, 2014; Phillips et al., 2015).

Recombination is initiated through the formation of double-stranded breaks (DSBs) in the DNA via the topoisomerase-like protein SPO11 (Neale and Keeney, 2006). DSBs can either be resolved as CO events, or non-crossover (NCO) events (Filippo et al., 2008). One of the primary explanations for the distal distribution of recombination events in barley comes from the cytological analysis of meiocytes using immunofluorescent staining techniques (Higgins et al., 2012). It was observed that synapsis of homologs initially occurs at the distal ends of chromosomes, progressing toward the centromeres with time. This spatiotemporal bias of synapsis allows DSBs to be formed preferentially at the distal ends of the chromosomes. The authors further hypothesized that crossover interference (Broman et al., 2002), the phenomenon in which one CO prevents further COs from forming in its vicinity, could be one of the factors that prevents further CO formation in the pericentromeric region (Higgins et al., 2012). In addition, it has been suggested that coordination between cycles of chromatin expansion/contraction and the formation of COs also contributes to this bias in distribution (Higgins et al., 2014).

Factors shown to influence meiotic recombination range from internal, genetic factors, such as the FANCM gene, which limits meiotic crossovers in *Arabidopsis* (Crismani et al., 2012), to external stresses such as soil magnesium content (Rey et al., 2018) and environmental temperature (Jain, 1957; Loidl, 1989). Temperature has recently been examined more thoroughly in barley with particular focus on its effect on the distribution of COs (Higgins et al., 2012; Phillips et al., 2015). Cytological analysis of meiocytes revealed a small but significant reduction in mean chiasma frequency between meiocytes grown at 22 and 30°C, as well as significantly more interstitial chiasmata for chromosome 5H when grown at 30°C compared to those grown at 22°C (Higgins et al., 2012). Further research attempted to expand on these results by utilizing SNP genotyping of a mapping population in addition to cytological analysis (Phillips et al., 2015). This method has the potential to provide a more precise evaluation of how large the shift in distribution of recombination events in response to temperature is, expanding on the categorical assignment of recombination events to either “distal” or “interstitial” positions in analysis of cytological data, as well as revealing the genes that are effected by a shift. Unfortunately, the authors did not perform a statistical analysis of the distribution of events in their SNP data, which was not made available for public use. In addition, they did not identify individual chromosomes in their cytological data (Phillips et al., 2015). It is therefore difficult to compare the two studies for consistency in results; these limitations of the literature provide the incentive to explore this topic further.

Existing research in wheat suggests that high temperatures prevent normal meiotic progression and therefore reduce the fertility of plants, with nullisomic lines identifying 5D as one of the chromosomes effecting temperature sensitivity (Draeger and Moore, 2017). However, there has yet to be a detailed analysis of the effects of temperature on recombination distribution in wheat. Here we have utilized a high-density SNP genotyping array (Allen et al., 2016) and four F2 mapping populations of an Apogee X Paragon (A × P) cross, each subjected to a different temperature during meiosis (10, 14, 26, and 28°C, respectively), to examine whether wheat behaves in the same way as barley, and to assess the utility of any shift in distribution to breeders.

## Materials and Methods

F1 seed from a Apogee × Paragon cross were obtained from Dr. Peter Jack at RAGT Seeds Ltd. These were randomly separated into four populations and grown initially in uniform conditions. Plants were grown in pots filled with peat-based soil and kept in a glasshouse at 15–25°C with 16-h light, 8-h dark. Plants were deemed to be undergoing meiosis when the base of the stem showed visible swelling due to the growth of the developing head within the flag leaf sheath, often referred to as the ‘booting’ stage of development (Barber et al., 2015). At this point, they were transferred to temperature-controlled cabinets at 10, 14, 26, and 28°C, respectively, for around 3 weeks. They were then transferred back to the glasshouse to avoid effects of temperature on pollen tube development. Seeds were then harvested from each of the populations. Leaf-tissue was harvested from F2 plants 12–14 days post-sowing, when the plants were at an early seedling stage. The sizes of the F2 populations were 80, 75, 70, and 78 individuals for temperature treatments of 10, 14, 26, and 28°C, respectively. DNA was extracted following the protocol in Pallotta et al. (2003) with minor modifications.

DNA concentration was assessed using a Qubit 2.0 Fluorometer and was the normalized to 23 ng/μl ready for analysis with the Axiom Wheat Breeder’s array (Allen et al., 2016). Sample preparation for array genotyping was performed with the Beckman Coulter Biomek FX. Samples were then genotyped using the Axiom 35K Wheat Breeders array in conjunction with the GeneTitan using standard Affymetrix protocols (Axiom 2.0 Assay for 384 samples P/N 703154 Rev. 2).

Axiom Analysis Suite (version 3.1.51.0) was used to assign genotype calls. Of the 6536 polymorphic SNPs between the two parental varieties present on the array, 2504 codominant SNPs of highest quality were selected through visual inspection of each cluster plot. Only markers with a clear delineation between genotyping clusters representing homozygotes for the Apogee allele, heterozygotes and homozygotes for the Paragon allele were used, and borderline markers were recoded as no-calls as a precaution against genotyping errors that could affect the recombination analysis. A custom R script was used to assign genotype calls to parental varieties, such that an “A” genotype represented an allele from Apogee, whilst a “B” genotype represented an allele from Paragon (Supplementary File 1).

Data from all four populations was amalgamated and used for initial genetic map construction using MultiPoint Complete (version 4.1). Markers exhibiting large segregation distortion (χ^2^ > 20), low informativity (LOD < 7), and large amount of missing data were removed from dataset before proceeding with genetic map construction. MultiPoint first performs binning of markers that have the same genotype across all individuals. These bins or “skeleton markers” are then clustered based on an initial threshold recombination fraction, in this case 0.2, which was iteratively increased up to a value of 0.34. After clustering, the markers were ordered in MultiPoint using a guided evolutionary strategy optimization algorithm (Mester et al., 2015) in conjunction with a jackknife resampling strategy to remove any markers that caused unstable regions in the marker order. Genetic distances of markers were estimated from recombination fractions using the Kosambi mapping function.

Only the skeleton markers in this initial genetic map were retained, as these are the only informative markers for evaluating recombination events. The cluster and ordering information from this initial genetic map was applied to the genotyping data from each of the four populations. Assignment of chromosomes to linkage groups was performed both by comparison of linkage groups to previous marker assignments based on nullisomic lines (Winfield et al., 2016), as well as BLASTN searches of probe sequences to the IWGSC RefSeq v1.0 assembly (IWGSC et al., 2018), hereafter referred to as the IWGSC assembly. In all cases, only BLAST hits with an *e*-value smaller than 10^–19^ were used. Four further genetic maps were then generated, one for each population, retaining the same clustering and ordering information from the first map, using the quickEst function in the R package ASMap (version 1.0–2) (Taylor and Butler, 2017). This allowed comparison of recombination distribution between temperature treatments using centimorgan values of markers. To verify the quality of the genetic maps, we performed a comparison with the Apogee X Paragon F5 map produced by Allen et al. (2016). As these maps both involve the same parental varieties, they should show close resemblance in their clustering of markers, and should also approach colinearity in their ordering of markers, allowing for small perturbations that may be caused by genotyping error or missing data (Hackett and Broadfoot, 2003; Wu et al., 2008).

Recombination events were detected by a change in genotype between consecutive markers in the genetic map. Transitions from a homozygous allele for one parent to a homozygous allele for the second parent were scored as two recombination events, as these would have required recombination to occur in this position in both of the gametes that formed the zygote. With the inference of recombination events from SNP data, genotyping error has the potential to erroneously inflate the number of events observed. The magnitude of this effect depends on both where the genotyping error occurs and what the erroneous assignment is, either homozygous or heterozygous. For instance, let us consider three markers used to genotype a single individual, M_1_, M_2_ and M_3_. If the genotypes of the markers are A, A, A (i.e., homozygous for the allele from the first parent) and M_2_ erroneously changes to B (i.e., homozygous for the allele from the second parent), in an F2 individual, this would indicate that four recombination events have occurred. In order to compensate for potentially erroneous genotypes, genotypes conforming to this three marker scenario where M_1_ and M_3_ are within 30 Mb of each other were recoded as no-calls.

To test statistically whether there was a shift in distribution of recombination events between treatments, it was necessary to take the mean distance of all recombination events within an individual from the centromere of the chromosome, which we will refer to henceforth as the mean recombination distance (MRD). Positions of centromeres were based on previously published ChIP-seq data for centromere specific histone 3 (CENH3) (IWGSC et al., 2018), whilst the position of each recombination event was taken as the midpoint between the two markers exhibiting the genotype change. Recombination events cannot be tested individually as events within a sample are not independent. For example, if two recombination events occur on the same chromosome during a single meiosis, the position of the second event is likely to be influenced by the position of the first due to crossover interference. In SNP data derived from mapping populations, individuals are a product of two meioses, one for each gamete that contributed to the formation of the individual. It is not possible to assign recombination events to one or the other meiosis, and it is therefore not possible to determine which events may have been influenced by crossover interference. For example, if we observe two events at 2% physical distance either side of the center, and a third event 10% physical distance from one of the telomeres, we know that it is very unlikely that the two central events occurred in the same meiosis, but we do not know which of them comes from the same meiosis as the distal event. Using individual recombination events then in our statistical analysis could cause conflation of the effects of the treatment, e.g., external temperature during meiosis, on recombination distribution with the effects of crossover interference.

In addition, since wheat chromosomes are not metacentric, with a mean ± SD centromere position of 40.5 ± 6.14% of the physical sequence for each chromosome (IWGSC et al., 2018), we partitioned our measurement of MRD between chromosome arms. Previous work suggests that crossover interference in wheat is strongest at distances smaller than 10 cM (Saintenac et al., 2009), which in addition to the distal distribution of recombination events in wheat, should preclude strong inter-arm effects of crossover interference. MRD was measured in units of percentage physical distance along chromosome arms.

The analysis of recombination distribution was performed using physical distances of markers along the IWGSC assembly as determined by BLAST. This allows us to relate shifts in distribution to genomic features such as genes. Wheat has a recombination distribution that is biased toward the telomeric regions of the chromosomes. This being the case, it was necessary to ensure that genetic map representations of the chromosomes had sufficient marker coverage to detect potential shifts away from the telomeric regions toward the centromeres. To do this, we defined anchor points along the chromosome at 0, 25, 50, 75, and 100 (% physical distance). We then identified the nearest marker to each of these anchor points. If the distance between any of the anchor points and their nearest markers was greater than 25, the chromosome was excluded from the analysis. So for example, if a chromosome consisted of markers at 40, 50, 60, 70, and 80%, it would be excluded as the nearest marker to the first anchor point (0), is 40, which is more than 25% away, precluding the analysis of recombination events at that end of the chromosome. In addition to ensuring adequate coverage, it was also necessary to ensure that the order of markers in the genetic maps and the physical map were concordant, as a discordant order could bias our analysis of recombination distribution. To do this, the longest increasing subsequence of physical positions of markers was taken for each chromosome, and all markers not included in this sequence were removed.

The data was also examined for other potential factors that could cause differences in MRD between temperature treatments. Genotyping error can be assessed with standard quality control variables produced by Axiom Analysis Suite, such as dish QC, which is based on the contrast between probe hybridization signals at non-polymorphic genome locations, and QC call rate, which measures the call rate of a subset of probes for a particular sample (Affymetrix, 2015). To assess whether genotyping error influenced recombination distribution, linear regressions were performed with these two variables using MRD as the response.

Another possible explanation for any observed shifts in MRD is that the sample sizes of the mapping populations used were too small. The distribution of recombination events may appear to be skewed toward the centromeres simply due to sampling error, i.e., if we happened to produce individuals that had more centromere-proximal events and were not observing the true value of MRD in the population. To test whether this was the case, we utilized simulated genotyping data from PedigreeSim (Voorrips and Maliepaard, 2012). This performs a detailed approximation of meiosis including the formation of bivalents, chiasmata, and meiotic divisions for a specified population structure. 200 genotyping datasets were produced, each containing an F2 mapping population of 500 individuals. These had a marker distribution based on chromosome 3B from the Apogee X Paragon F2 genetic map produced here. Further datasets with sample sizes of 250, 100, and 30 individuals were then created by randomly subsetting the original 200 datasets. For each sample size, 100,000 random combinations of four datasets were generated. We then performed a Kruskal–Wallis test on MRD across the whole chromosome, for every combination, for every sample size, examining whether there was an increasing number of significant tests as sample size decreased, which would indicate that sample size has an influence on MRD.

## Results

**FIGURE 1:**
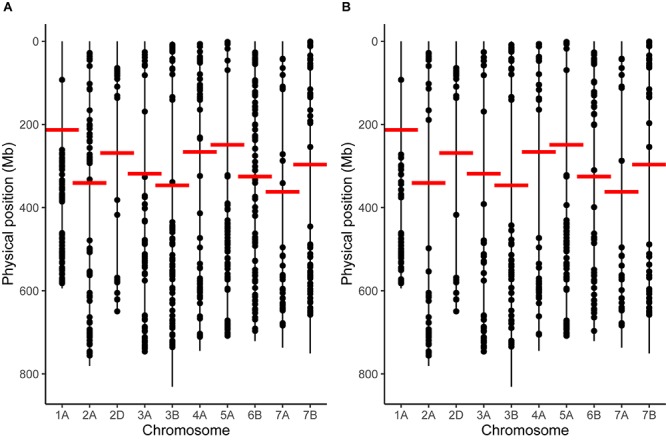
Marker distribution for chromosomes that passed our filtering criteria. **(A)** Marker distribution before removal of markers with discordant order between genetic and physical maps via the longest increasing subsequence. **(B)** Marker distribution after removal. Vertical lines represent the entirety of the length of each chromosome, taken from the IWGSC assembly, whilst points represent the positions of markers. Horizontal red lines mark the position of the centromere on each chromosome.

To analy**z**e potential changes in recombination distribution between temperature treatments, it was first necessary to select only the chromosomes that had a sufficient marker distribution to detect an inward shift in recombination distribution. We found that chromosomes 1A, 2A, 2D, 3A, 3B, 4A, 5A, 6B, 7A, and 7B met our criteria for marker distribution (Figure 1). These chromosomes contained a total of 1394 markers in the initial genetic map prior to any filtering. 870 of these markers had valid BLAST hits to the IWGSC assembly and could therefore be assigned physical positions (Figure 1A); the remaining markers were discarded. Markers were then filtered further: any markers that had a discordant order between the genetic and physical maps were removed (Figure 1B), leaving 442 markers, or 44.2 ± 12.15 (mean ± SD.) markers per chromosome for further analysis (Supplementary File 2). We will refer to this map as the filtered genetic map. Overall marker distribution was generally preserved during this stage, although the number of markers immediately adjacent to the centromere for chromosomes 2A and 6B was noticeably reduced (Figure 1B). The mean ± **SD** distance between markers (Mb) was 8.9 ± 26.4, 17.33 ± 48.5, 24.39 ± 62.64, 20.58 ± 42.2, 15.78 ± 44.34, 10.71 ± 33.35, 13.4 ± 22.58, 20.69 ± 46.89, and 17.3 ± 39.21 for chromosomes 1A, 2A, 2D, 3A, 3B, 4A, 5A, 6B, 7A, and 7B, respectively. Marker density was generally highest at the distal ends of the chromosomes with a drop in the number of markers in regions surrounding the centromeres (Figure 1).

**FIGURE 2:**
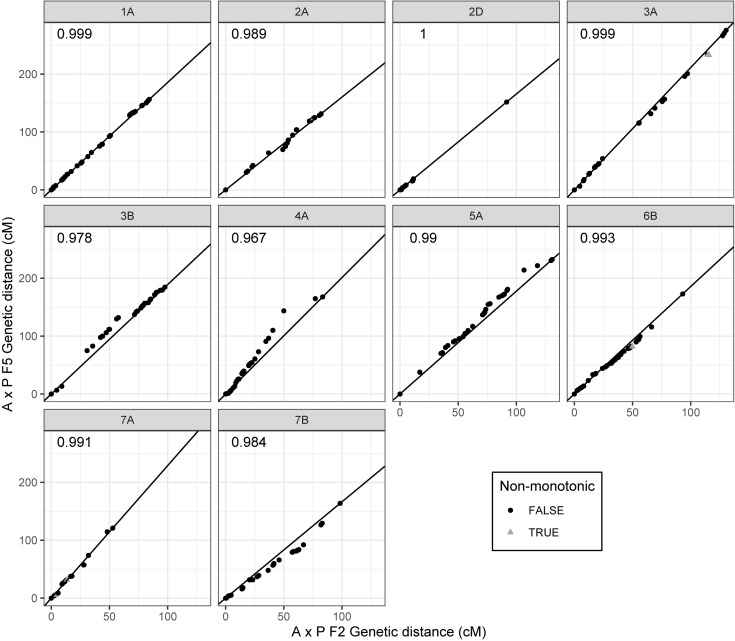
Comparison of marker order and distribution between the filtered F2 Apogee × Paragon genetic map and the F5 genetic map produced by Allen et al. (2016). Points represent markers and their genetic positions (cM) in the respective maps. Some of the markers present in the F2 map are not present in the F5 map due to a difference in marker selection procedure between studies. Centimorgan values have therefore been normalized such that map comparisons start at zero whilst retaining inter-marker distances. Deviations from the diagonal line represent differences in the recombination distribution between maps; perfect adherence to the line represents complete coherence between maps in both marker order and marker distribution. Markers that have an inverted order between maps (markers deviating from monotonicity) are represented as gray triangles, whereas markers that are consistent in order represented as black circles. *R*^2^ values of linear regressions of the F5 position as a function of the F2 position are shown in the upper left corner of each plot. Chromosomes are labeled in gray panels above each plot.

Of the 2503 markers selected from Axiom Analysis Suite for the F2 A × P genotyping data produced here, 1563 were present in the final F5 A × P genetic map produced in Allen et al. (2016). For comparison to the F5 A × P map, the filtered F2 map was used due to its importance in subsequent analyses such as assessing MRD. Clustering between genetic maps was highly consistent; none of the linkage groups from the F2 map contained markers that were present in a linkage group that was identified as a different chromosome in the F5 map (Table 1). To test the concordance of marker distribution and order between maps, linear regressions for each chromosome were performed predicting the genetic position (cM) of a marker in the F5 map based on the position of the marker in the F2 map. All were highly significant (*p* < 10^–16^ for all chromosomes after Bonferroni correction), with over 96% of the variation in the F5 position being explained by the F2 position for every chromosome tested (Figure 2). Chromosomes 1A, 2D, 3B, 4A, 5A, and 7B had perfectly colinear marker bins between maps. In the six cases where marker order did differ between maps, in chromosomes 2A, 3A, 6B, and 7A, it did so in adjacent pairs of markers contained within very small centimorgan windows: the mean ± SD distance (cM) between these inverted pairs of markers was 0.33 ± 0.25 cM. This was expected; it is well known that small genetic distances between markers can influence map ordering algorithms (Hackett and Broadfoot, 2003).

**TABLE 1:** Comparison of clustering of markers between the F2 Apogee × Paragon genetic map generated here and the F5 map generated previously.

**A × P F2 LG**	**A × P F5 LG 1**	**A × P F5 Num. Markers 1**	**A × P F5 LG 2**	**A × P F5 Num. Markers 2**	**A × P F5 LG 3**	**A × P F5 Num. Markers 3**	**Not present**
1A	1A	44	–	–	–	–	12
2A	2A	24	–	–	–	–	19
2D	2D	12	–	–	–	–	13
3A	3A	28	–	–	–	–	8
3B	3B	35	–	–	–	–	12
4A	4A	34	–	–	–	–	12
5A	5A	34	5A2	11	–	–	22
6B	6B	39	–	–	–	–	12
7A	7A	19	7A3	2	–	–	11
7B	7B	27	–	–	–	–	12

**The first column indicates the linkage group/chromosome from the F2 genetic map, whereas the subsequent “LG” columns indicate linkage groups that share markers with this F2 linkage group. Columns labeled “Num. markers” indicate the number of markers shared between linkage groups. The final column indicates the number of markers in the F2 linkage group that were not present in the F5 genetic map*.*

**FIGURE 3:**
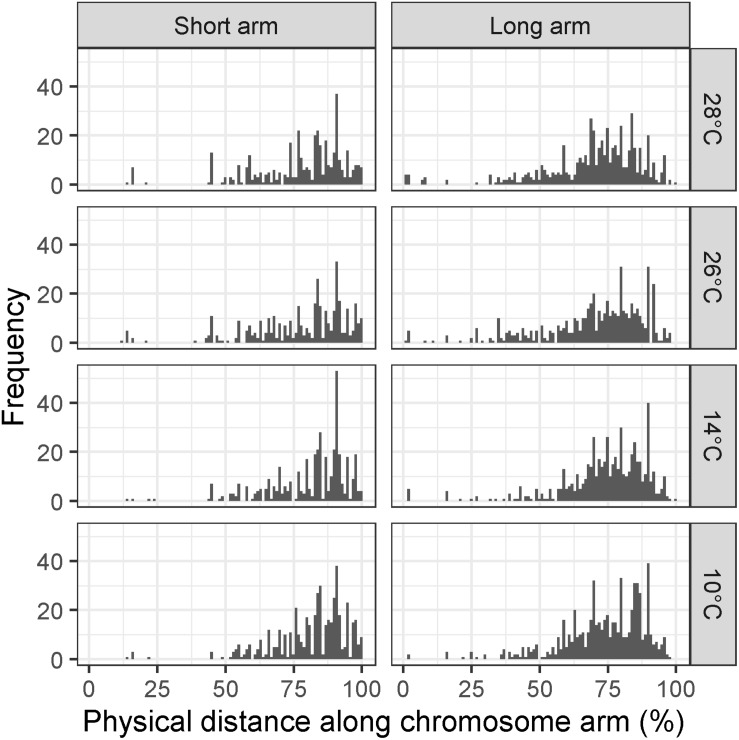
Histograms of mean recombination distance (MRD) in Apogee × Paragon F2 populations across all chromosomes for temperature treatments of 10, 14, 26, and 28°C, respectively. Recombination is measured as the mean distance of recombination events from the centromere of the chromosome in each individual plant to avoid conflation of crossover interference with temperature treatment. Panels on the left show MRD for all short chromosome arms, whereas panels on the right show MRD for all long chromosome arms.

To test whether there was a significant difference in the distribution of recombination events between temperature treatments, the MRD (see section “Materials and Methods”) was calculated for each individual in each treatment. We first examined MRD for all chromosomes at once to see if there was a genome-wide effect of temperature on recombination distribution. In the Apogee × Paragon F2 populations, a Kruskal–Wallis test of the MRD in the long arms of all chromosomes reveals a highly significant difference between all four temperature treatments (10, 14, 26, and 28°C) (χ^2^ = 25.63, d.f. = 3, *p* < 0.0001). Likewise, this test was highly significant for the short arms (χ^2^ = 12.13, d.f. = 3, *p* < 0.007). Temperatures 10 and 14°C showed evidence of a more distal distribution of recombination events compared to 26 and 28°C, with mean ± SD MRD values (units are percentage of chromosome arm) of 73.7 ± 15.2, 74.34 ± 15.56, 70.19 ± 18.75, and 70 ± 18.03, respectively, for long chromosome arms (Figure 3), and values of 81.48 ± 13.6, 81.19 ± 14.24, 77.87 ± 17.52, and 77.99 ± 16.69 for short chromosome arms (Figure 3).

To examine whether any one treatment was exerting a strong influence on the Kruskal–Wallis test of MRD, individual treatments were removed before performing the test again. MRD remained significantly different between temperature treatments when any of the individual treatments were removed before the test (*p* < 0.005 for all temperatures in the long arm; *p* < 0.05 for all temperatures in the short arm). We then removed pairs of treatments before performing the test again to examine whether high and low temperature treatments were clustered in their effect on the test (Table 2). For the long chromosome arms, the test only became insignificant when either both low temperature treatments (10 and 14°C) were removed (χ^2^ = 0.5, d.f. = 1, *p* = 0.48) or when both high temperature treatments (26 and 28°C) treatments were removed (χ^2^ = 0.75, d.f. = 1, *p* = 0.37). This was also the case for the short chromosome arms (Table 2).

**TABLE 2:** Examining the effect of removal of pairs of temperature treatments before performing the Kruskal–Wallis test on differences in mean recombination distance (MRD).

**Treatments removed**	***p*-value (long arm)**	**χ^2^ (long arm)**	**Bonferroni corrected *p*-value (long arm)**	***p*-value (short arm)**	**χ^2^ (short arm)**	**Bonferroni corrected *p*-value (short arm)**
10°C, 14°C	0.44727	0.57757	1	0.76677	0.08797	1
10°C, 26°C	**0.00001**	19.45922	**0.00006**	**0.00627**	7.471	**0.03762**
10°C, 28°C	**0.00094**	10.94939	**0.00562**	**0.02589**	4.96322	0.15535
14°C, 26°C	**0.0003**	13.06434	**0.00181**	**0.00884**	6.85392	0.05307
14°C, 28°C	**0.00974**	6.68248	0.05842	**0.02821**	4.81548	0.16923
26°C, 28°C	0.37159	0.79835	1	0.7525	0.09944	1

**Significant p-values are highlighted in bold. Bonferroni corrections were performed within chromosome arms.**

In addition to the genome-level analysis of recombination distribution, we also tested for differences in MRD between temperature treatments for individual chromosomes, which differed in their response to changes in temperature during meiosis. The long arms of chromosomes 1A, 3B, as well as the short arms of chromosomes 2A and 7A showed significant differences in MRD between all temperature treatments as determined by a Bonferroni-corrected Kruskal–Wallis test (Table 3). The long arm of chromosome 1A was most significant, followed by the short arm of 2A (Table 3).

**TABLE 3:** Results of Kruskal–Wallis test for difference in MRD between temperature treatments for individual chromosomes.

**Chromosome**	***p*-value (long arm)**	**χ^2^ (long arm)**	***p*-value (short arm)**	**χ^2^ (short arm)**	**d.f.**
1A	**0.00003**	28.6104816	–	–	3
2A	0.21645	9.6644259	**0.00032**	23.2965561	3
2D	1	0.5983921	1	1.0994944	3
3A	1	4.9709325	0.88841	6.2809669	3
3B	**0.04108**	13.2592939	0.98446	6.0461797	3
4A	1	6.1989495	0.33398	8.4774506	3
5A	0.38483	8.3968958	1	2.9122176	3
6B	1	5.255726	1	0.5728396	3
7A	0.33544	8.7008418	**0.00485**	17.5702203	3
7B	1	1.9496573	1	1.7397095	3

**P-values have undergone a Bonferroni correction for multiple testing within chromosome arms. The short arm of 1A is not included as the marker distribution was not sufficient.* Significant *p*-values are highlighted in bold.*

**FIGURE 4:**
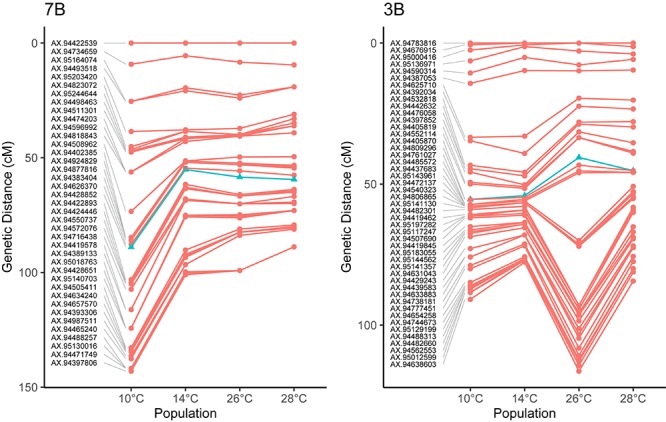
Comparisons of genetic maps for four different temperature treatments in Apogee × Paragon F2 populations. Noticeable in on chromosome 3B is a large expansion of the central regions between treatments, for example between temperatures 14 and 26°C on chromosome 3B, indicating the more recombination events occurred in these regions at higher temperatures. In other chromosomes, the total amount of recombination decreases at higher temperature treatments, such as in 7B. These differences highlight the fact that temperature does not act equally on all chromosomes during meiosis. Markers closest to the centromere [the location of which are taken from IWGSC et al. (2018)] are highlighted as blue triangles, for chromosome 3B, this marker is AX-94405870, located within 100 Mb of the centromere, whilst for chromosome 7B, this marker is AX-94428852, located within 50 Mb of the centromere.

To investigate these chromosome-level differences in recombination distribution between temperature treatments further, we compared the centimorgan distribution of markers between genetic maps. Examination of the chromosome 7B, which was the least-significant chromosome in our MRD analysis, showed a reduction in the number of recombination events between temperatures 10 and 14°C, with little change in the distributions of events between 14, 26, and 28° treatments (Figure 4). Chromosome 3B on the other hand had a similar distribution of recombination events between temperatures 10 and 14°C, before expanding in central regions of the genetic map between temperatures 14 and 26°C (Figure 4).

**FIGURE 5:**
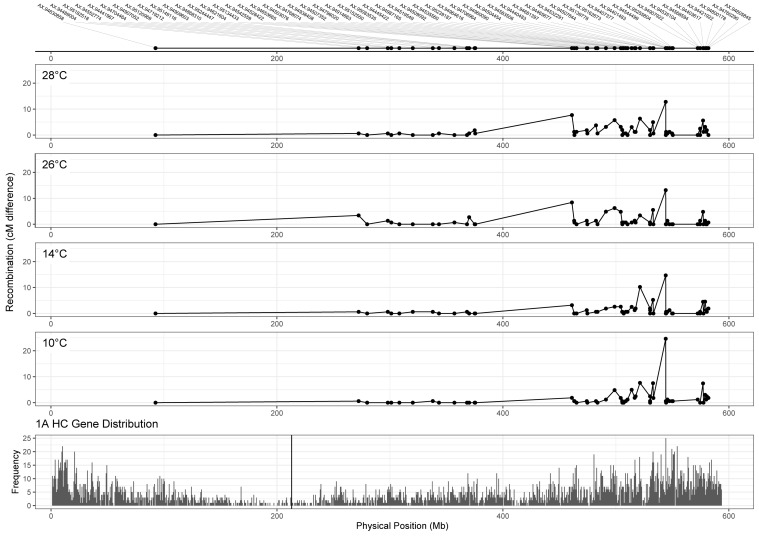
Comparison of recombination distribution between temperature treatments for chromosome 1A. Marker names are indicated in the top panel. Panels 2–5 show recombination maps for Apogee × Paragon populations that have undergone meiosis at different temperatures. Recombination is measured as the difference in centimorgans between adjacent markers. The bottom panel shows the distribution of high confidence genes along chromosome 1A of the IWGSC v1.0 wheat genome assembly, with the centromere marked by the vertical black line. Higher temperature treatments (26 and 28°C) seem to induce recombination in gene-rich areas nearer the centromere at around 461 Mb.

Whilst we can detect differences in MRD between temperature treatments for some chromosomes, it is also important to assess the potential utility of this difference to wheat breeders in terms of its relation to gene distribution. To do this, we compared chromosome 1A recombination distributions for each treatment (Figure 5, top four panels), to the distribution of genes along the chromosome (Figure 5, bottom panel). Higher temperature treatments of 26 and 28°C appear to induce recombination in regions closer to the centromere from 375 to 461 Mb (markers AX-94621604 and AX-95134433), with a difference of 8.4 and 7.7 cM between markers in 26 and 28°C, respectively, compared to 1.87 and 3.19 cM in 10 and 14°C treatments. Despite these differences in recombination distribution between treatments, many genes remained highly linked regardless of temperature, for example from 280 to 350 Mb (Figure 5).

Another area of interest was the potential presence of temperature-dependent recombination hotspots, defined here as regions that contained recombination events in the two high temperature treatments, that also lacked recombination events in both lower temperature treatments. There was evidence of these hotspots on chromosomes 1A, 2A, 2D, 3A, 3B, 4A, and 6B (Table 4). The inter-marker areas of these hotspots contained a total of 868 genes. The hotspot with the largest disparity in number of recombination events between temperatures was found on chromosome 3B, spanning from 485.69 to 488.53 Mb, with differences in cM values between flanking markers of 0, 0, 24.04, and 4.95 at temperatures of 10, 14, 26, and 28°C, respectively. Inspection of the genotype data revealed that these recombination events were not double events, and visual examination of the SNP cluster plots for both flanking markers revealed clearly delineated genotype clusters in both cases, indicating that genotyping error was unlikely to be the cause of this difference. Annotations for the 19 genes within these two makers include DNA-directed RNA polymerase III subunit RPC3, 50S ribosomal protein L15 (putative), Ubiquitin-conjugating enzyme E2, NAC domain protein and an Auxin-responsive protein. The second most notable hotspot was on chromosome 1A, where the 26 and 28°C treatments have differences of 2.7 and 0.68 cM, respectively, between markers AX-94909603 and AX-94868310 at 370 Mb (Figure 5).

**TABLE 4:** Regions containing potential temperature-dependent hotspots, defined as having recombination events in both high temperature treatments, whilst lacking recombination events in both low temperature treatments.

**Chromosome**	**Marker before**	**Marker after**	**Physical position 1st marker**	**Physical position 2nd marker**	**Phys difference**	**10°C cM difference**	**14°C cM difference**	**26°C cM difference**	**28°C cM difference**	**Number of genes**	**Distance from centromere**
3B	AX.94485572	AX.94437683	485.69	488.53	2.84	0	0	24.04	4.95	19	140.71
1A	AX.94909603	AX.94868310	368.2	370.1	1.9	0	0	2.7	0.63	28	156.15
2D	AX.95199672	AX.94641475	73.59	74.94	1.35	0	0	1.42	1.31	16	−194.19
1A	AX.95255804	AX.94907922	572.35	574.48	2.13	0	0	1.36	2.47	37	360.42
3A	AX.94606333	AX.95008057	479.78	485	5.22	0	0	1.36	0.6	40	163.99
4A	AX.95111935	AX.94478215	140.71	164.4	23.69	0	0	1.36	1.89	117	−113.45
3A	AX.95164207	AX.94393045	168.84	391.49	222.65	0	0	1.34	0.62	508	−38.24
2D	AX.94482198	AX.94457291	620.27	621.07	0.8	0	0	0.7	0.66	20	352.22
6B	AX.94451495	AX.94896306	474.22	476.72	2.5	0	0	0.69	1.23	18	150.22
6B	AX.95123143	AX.94403686	549.19	555.95	6.76	0	0	0.68	0.62	44	227.32
1A	AX.94445422	AX.94987165	505.68	506.85	1.18	0	0	0.68	0.64	11	293.27
6B	AX.94946141	AX.95110233	562.48	563.06	0.58	0	0	0.67	0.62	3	237.52
2A	AX.94514944	AX.94536561	605	605.18	0.18	0	0	0.67	0.64	7	264.39

**Also shown are the number of high-confidence genes within the marker interval from the IWGSC assembly annotation. Negative distances from the centromere indicate that the hotspot occurs on the short arm of the chromosome.**

**FIGURE 6:**
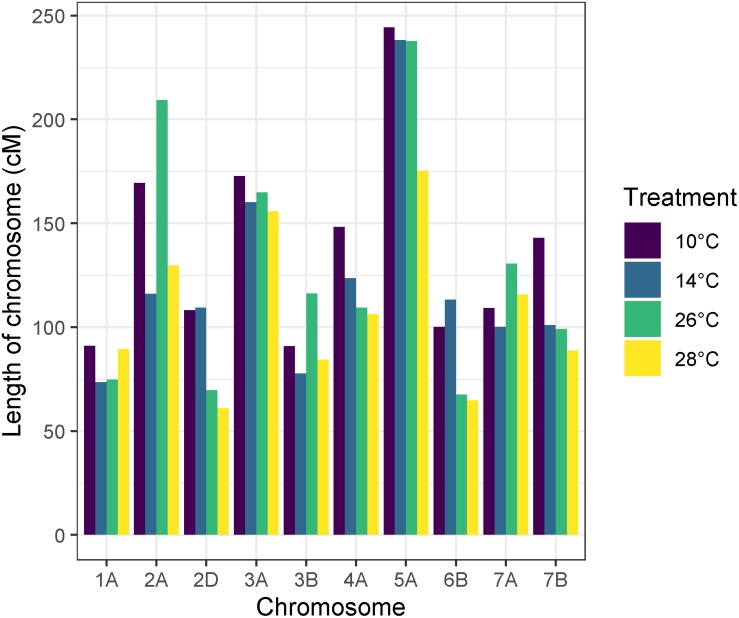
Apogee × Paragon genetic map lengths by chromosome across temperature treatments. Chromosome 5A has the largest map length and therefore the highest number of recombination events in all temperature treatments. In some chromosomes, higher temperature treatments have less recombination events overall, such as in chromosome 2D, 6B, and 7B.

**FIGURE 7:**
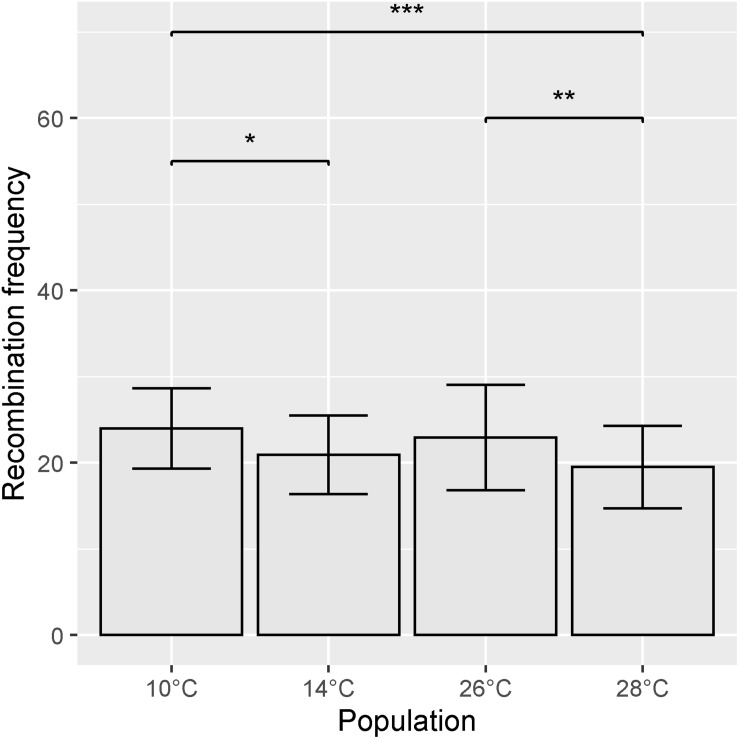
Mean recombination frequency across all chromosomes for each temperature treatment. Error bars represent ± SD from the mean. Significantly different populations are indicated by asterisks (*p < 0.05, **p < 0.01, ***p < 0.001).

Recombination frequency varied between temperature treatments, with total map lengths of 1377.43, 1213.2, 1279.8, and 1071.65 cM for 10, 14, 26, and 28°C degree treatments, respectively. Chromosome 5A had the highest number of recombination events in all temperature treatments (Figure 6). An ANOVA of recombination frequency in individuals across all chromosomes between populations revealed significant differences between temperature treatments 10 and 14°C (*p* < 0.0001), 10 and 28°C (*p* < 0.00001) as well as 26 and 28°C (*p* < 0.001) as determined by a Tukey *post hoc* test (Figure 7). The frequency of recombination events follows a U-shaped pattern between temperatures 10, 14, and 26°C, before declining between 26 and 28°C (Figure 7). The mean ± SD number of recombination events across all individuals was 21.83 ± 5.32.

Genotyping error was found not to influence MRD. Linear regressions of standard sample quality control metrics [either dish quality control (DQC) or quality control call rate] as the explanatory variable and MRD as the dependent variable for each chromosome were all none-significant. *R*^2^ values for each chromosome were all smaller than 0.026, meaning that less than 2.6% of the variation in MRD was explained by these variables in every case.

In addition, the simulation experiment indicated that sample size does not influence MRD either. For sample sizes of 500, 250, 100, and 30, 6.29, 3.01, 7.15, and 2.03% of the 100,000 combinations of samples exhibited significant differences in MRD, as shown by a Kruskal–Wallis test. If sample size had an effect, we would expect these percentages to show a consistent trend, i.e., increasing or decreasing with sample size. It should also be noted that these percentages are close to the expected number of false-positives (5%) at this alpha threshold (0.05).

## Discussion

The data presented here is the first detailed analysis of the effect of environmental temperature during meiosis on the distribution and frequency of recombination events in wheat. Our data, based on high-density SNP genotyping of four Apogee × Paragon mapping populations, each subjected to a different temperature during meiosis, reveal a clear effect of temperature on the distribution of recombination events. This effect is visible, although subtle, in Figure 3, where lower temperature treatments appear to have a more distal distribution of events in both short and long arms of the chromosomes. In Figure 3, there are some MRD values that appear to be within 25% of the centromere, which contrasts with evidence from previous studies (Saintenac et al., 2009; Choulet et al., 2014) that the distribution of recombination in wheat is limited to the distal ends of the chromosomes. These MRD values are most likely are artifacts of the method used here to measure recombination, where the position of recombination is assigned as the midpoint between two markers, in conjunction with the reduced marker density in centromeric regions after filtering (Figure 2). This should not have any impact on the analysis of the relative difference in recombination distribution between temperature treatments, as all treatments used the same genetic map with the same distribution of markers. The statistical analyses of MRD for individual chromosomes show that the effect of temperature on recombination distribution is limited to specific chromosomes/chromosome arms, suggesting that chromosomal structure may influence the susceptibility of chromosomes to changes in temperature. This mirrors results from barley (Higgins et al., 2012; Phillips et al., 2015).

A comparison of the Apogee × Paragon F2 genetic map produced here to the F5 genetic map of the same cross from Allen et al. (2016) shows a high degree of similarity in the clustering (Table 1), ordering (Figure 2) and genetic distribution (Figure 2) of markers, indicating that the genetic map is robust. Noticeable in the comparison of clusters, however, is that some of the chromosomes in the F5 map, such as 5A and 7A, are split into multiple linkage groups. This could explain the markers that are present in our map that are not present in the map of Allen et al. (2016), as these were most likely resolved as smaller linkage groups in the latter, which were then discarded. Our marker selection process was more stringent than in Allen et al. (2016) as we only used codominant markers that were categorized as “Poly high resolution,” the highest quality marker categorization in Axiom Analysis Suite (Bassil et al., 2015), whereas multiple categories of marker were used in the map of Allen et al. (2016). In addition, in this study the SNP cluster plots for each marker were visually inspected and only markers with a clear delineation between genotyping clusters representing homozygotes for the Apogee allele, heterozygotes and homozygotes for the Paragon allele were used.

Our confidence in the validity of the results is increased by the fact that differences in MRD between treatments only become non-significant when two treatments are removed, either both of the low temperature treatments (10 and 14°C) or both of the high temperature treatments (26 and 28°C). This effect was less pronounced in the short arms of chromosome, indicating that perhaps temperature has less of an effect on these regions. However, this could also simply be the result of lower marker density in short chromosome arms. It is clear the observed shifts in recombination distribution are not due to genotyping error, as the results of linear regressions of standard quality control metrics against MRD were all non-significant. Furthermore, the results of the simulation experiment show that the differences in MRD observed in the Apogee × Paragon crosses were not the result of sampling error due to small population size.

In order to perform a statistical analysis on the differences in distribution of recombination events between temperature treatments, it was necessary to associate genetic maps to the physical wheat genome assembly. To do this we devised a method that utilizes the longest increasing subsequence of BLAST positions of marker sequences to the IWGSC assembly, allowing the measurement of MRD in each individual. This unfortunately comes with a caveat, in that markers that do not conform to this sequence must be removed, which reduces the density of markers in certain regions (Figure 1). This is one of the primary limitations of using MRD and means that our ability to precisely localize certain recombination events is reduced. The reason for the reduction in marker density is likely due to structural differences between varieties Apogee and Paragon, used here to generate the genetic maps, and Chinese Spring, the only wheat variety currently to have a completely publicly available, chromosome-level genome assembly. A reanalysis of the data could provide further information should a chromosome-level genome assembly of either Apogee or Paragon be released in the future. Despite these caveats, the data has sufficient marker density to inform our picture of recombination distribution in many chromosomal regions (Figure 1). The process used in this study conforms closely to the process that a wheat breeder might implement in their development of new varieties, and so has direct bearing on the applications of temperature in wheat.

In addition to the effect of temperature on the distribution of recombination events, we also observed an effect on the frequency of recombination events. Recombination frequency followed a U-shaped response from 10° to 26°C (Figure 7), which is consistent with results in *Arabidopsis* presented by Lloyd et al. (2018). This is followed by a dip at 28°C, which differs from the results in *Arabidopsis*. Lloyd et al. (2018) suggest that this effect is primarily produced by changes in class I interfering crossovers and speculate that recombination may be minimized in organisms that are already well-adapted to their environment and living in optimal conditions.

The number of recombination events that occurred in each bivalent can be estimated by dividing the mean number of recombination events across all individuals by the number of chromosomes, then dividing that by two (as each zygote is composed of two gametes). This gives us an estimate of 1.09 recombination events per bivalent. This is lower than published counts of chiasmata per bivalent in wheat, which are around 2.3 (Miller and Reader, 1985). There are several factors that contribute to this underestimation. Firstly, double recombination events that occur in between marker positions are not detectable using SNP data. In addition, in a sequence of heterozygous genotypes, if both gametes have a recombination event between the same two markers, the recombination event will not be detected. Finally, in some of the chromosomes analyzed, there were a lack of markers at the very distal ends of the chromosomes (Figure 2), and so recombination events that occurred in these regions could not be detected.

Our data indicate that increasing the temperature during meiosis could have some limited use to breeders in breaking up centromeric linkage blocks. We observed four chromosome arms with significant shifts in recombination distribution between temperature, and several putative temperature-dependent recombination hotspots (Table 4). Included in the 3B temperature-dependent hotspot were putative genes influencing plant development, including a NAC domain protein (Puranik et al., 2012) as well as an auxin-responsive protein (Teale et al., 2006). Despite these points, there are still many genes that remain highly linked in regions closer to the centromere on many chromosomes. To achieve thorough mixture of these genes in progeny of crosses, it will be important for breeders to explore other avenues of manipulating recombination distribution. One potential option is to produce fusion proteins linking SPO-11, the protein that initiates recombination through production of DSBs in the DNA, to other DNA targeting proteins, such as Zinc finger elements, Transcription activator-like elements or dead Cas9. Research in yeast using these methods revealed a 2.3- to 6.3-fold increase in COs near targeted regions (Sarno et al., 2017). Applications of this technique to agriculture outside of research could, however, be limited due to increasingly strict legislation, such as the 2018 European Union ruling in case C-528/16 that organisms edited using directed mutagenesis methods such as CRIPR-Cas9 will be officially classified as genetically modified organisms.

If breeders do decide to utilize temperature as a means of altering recombination distribution, they will also have to consider the loss in fertility of wheat plants at higher temperatures of meiosis (Draeger and Moore, 2017). These elevated temperatures would therefore need to be employed strategically and transiently in between generations that are grown at lower temperatures. It would be of interest to examine the epigenetic effects of elevated temperature during meiosis, such as whether there are any lasting changes inherited by progeny, and whether these changes are detrimental to plant growth.

In conclusion, the work here shows that temperature has a subtle effect on the frequency and distribution of recombination events in wheat. The analysis of recombination distribution in comparison to gene distribution indicates that the utility of this effect may be limited, with large amounts of genes remaining under strong linkage, and thus inaccessible to manipulation by breeders. Future work involving cytological analysis of wheat meiocytes subjected to different temperatures would be of interest, as the principle of consilience asserts that confidence in a result increases when reached independently through different methods.

## Data Availability Statement

The data associated with this study have been deposited to EVA: Project: PRJEB36318, 10° analysis: ERZ1284837, 14° analysis: ERZ1284835, 26° analysis: ERZ1284838, and 28° analysis: ERZ1284836.

## Author Contributions

AC planted and harvested F2 seed, performed DNA extraction, performed analysis of data, and wrote the manuscript. AB performed genotyping. KE conceived of the idea for the project, grew F1 seed, and performed temperature shock treatments. KE and AB edited the final manuscript.

## Conflict of Interest

The authors declare that the research was conducted in the absence of any commercial or financial relationships that could be construed as a potential conflict of interest.
